# Bojungikgitang and banhabaekchulchonmatang in adult patients with tinnitus, a randomized, double-blind, three-arm, placebo-controlled trial - study protocol

**DOI:** 10.1186/1745-6215-11-34

**Published:** 2010-03-28

**Authors:** Nam-Kwen Kim, Dong-Hyo Lee, Jung-Hun Lee, Yong-Leol Oh, In-Hwan Yoon, Eun-Sung Seo, Chung-Ho Lee

**Affiliations:** 1 Opthalmology Otolaryngology & Dermatology, Wonkwang University, Sanbon Oriental Medical Center, Gunpo, Korea; 2 Otolaryngology, Wonkwang University, Sanbon Medical Center, Gunpo, Korea; 3 Gastroenterology, Wonkwang University, Sanbon Medical Center, Gunpo, Korea; 4 Department of Oriental Medicine, Graduate School of Wonkwang University, Iksan, Korea

## Abstract

**Background:**

Tinnitus is the perception of hearing a sound for which there is no external acoustic source. It is often associated with sudden, temporary hearing loss and has a clear impact on a patient's quality of life. Despite numerous trials, there are no treatments that can be considered well established in terms of providing replicable long-term tinnitus reduction. Complementary and alternative medical approaches have been employed to relieve symptoms of tinnitus. Bojungikgitang and banhabaekchulchonmatang are among the most strongly preferred and widely used herbal medicines for tinnitus in Korea, as they cause very few serious adverse effects.

We aim to establish basic clinical efficacy and safety data for bojungikgitang and banhabaekchulchonmatang, which are approved as herbal medications by the Korea Food and Drug Administration in adult patients with tinnitus.

**Methods/Design:**

This study was a randomised, double-blind, placebo-controlled trial with three parallel arms (bojungikgitang, banhabaekchulchonmatang, and a placebo). Participants included in the study met the following criteria: typical conditions of intermittent or continuous tinnitus, for more than three months, with involuntary perceptions of the concept of a sound in the absence of an external source. Participants received bojungikgitang, banhabaekchulchonmatang, or a placebo-drug for eight weeks. The total duration of each arm was eleven weeks. Each participant was examined for signs and symptoms of tinnitus before and after taking medication. Post-treatment follow-up was performed two weeks after the final administration of medication.

**Discussion:**

This trial provided evidence for the efficacy and safety of bojungikgitang and banhabaekchulchonmatang in adult patients with tinnitus. The primary outcome measure was the Tinnitus Handicap Inventory, an assessment used to identify difficulties that may be experienced due to tinnitus. The secondary measures were included an Acoustic Examination and the Visual Analogue Scale. We employed the Euro-Qol 5-Dimension and the Health Utilities Index Mark 3, a health-related quality of life questionnaire. Safety was assessed by complete blood cell count, erythrocyte sedimentation rate, blood chemistry, urine analysis, PA chest film, brain computed tomography, otologic examination, and vital signs.

**Trial registration:**

Current Controlled Trials ISRCTN23691284

## Background

Tinnitus is a common disorder,[1] with approximately 5-15% of the general population experiencing an unremitting sensation of tinnitus [2]. Of patients who suffer with the condition, 1 to 2% are seriously impaired. Hearing loss, regardless of the cause, is the most important risk factor for the development of tinnitus. The condition can be severe enough to affect quality of life, result in sleep disturbances, cause work impairment, and lead to psychiatric distress in 1-3% of the general population [3].

Despite considerable research, there is no generally effective treatment for people who suffer from tinnitus [4]. Attempts at pharmacological treatment of patients who have tinnitus have proven to be disappointing. No single drug has been shown to be effective in appropriately controlled clinical trials [5,6]. There are also no drugs approved by the United States Food and Drug Administration (FDA) or the European Medicine Agency for the treatment of tinnitus [7]. Recent pathophysiological investigations have provided convincing evidence that tinnitus is associated with abnormal activity in the central auditory system, with plastic transformations of the central auditory system, and with abnormal links between the auditory and other seemingly non-related neural systems. This degree of complexity may explain why no single drug has been shown to be effective in the treatment of tinnitus [6].

In this context, various complementary and alternative medicine (CAM) treatments have been administered for tinnitus in clinical practice. Some studies have reported the effectiveness of acupuncture or herbal medicine, which is the most popular form of CAM therapy, for the treatment of tinnitus when used alone or concomitantly with usual care [8-11]. In the few number of trials that have been conducted, the quality and the small sample sizes of these studies make it difficult to reach firm conclusions concerning these treatments. Well-designed randomised controlled trials (RCTs) are needed to examine the efficacy of treatments for tinnitus.

The purpose of this study was to establish the basic clinical efficacy and safety data for bojungikgitang and banhabaekchulchonmatang, which are approved as herbal medications by the Korea Food and Drug Administration (KFDA) in patients with tinnitus. Because of the absence of a gold-standard for the treatment of tinnitus, this trial will be conducted to detect the effectiveness of these herbal medicines according to comparisons to a placebo-controlled group.

The use of these herbal medicines for tinnitus was based on the principles of Traditional Korean Medicine (TKM). In this medical tradition, tinnitus usually results from irregularities in bowel and visceral (zang-fu) functioning. In TKM, bojungikgitang is administered to treat the pattern of qi-deficiency and banhabaekchulchonmatang is used to treat the gallbladder deficiency associated with tinnitus [8]. Both bojungikgitang and banhabaekchulchonmatang have disease codes related to tinnitus in the Korean National Health Insurance Data (KNHID), and they can be fully covered by Korean National Health Insurance (KNHI).

We conducted a randomised, double-blind, three-arm, placebo-controlled trial of bojungikgitang and banhabaekchulchonmatang in adult patients with tinnitus.

## Methods/Design

**Figure 1:**
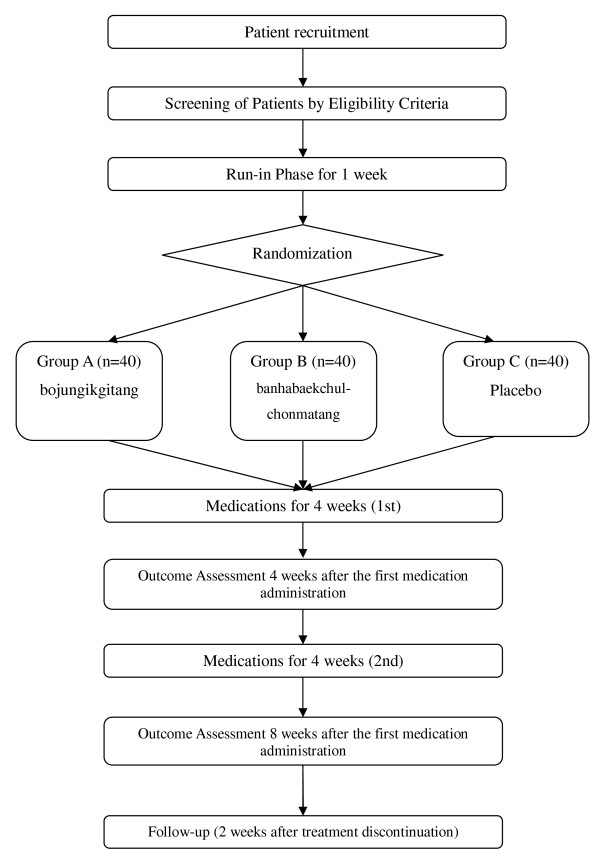
**Study Flow Chart**.

This study was a randomised, double-blind, three-arm, placebo-controlled trial. Participants fulfilling eligibility criteria were selected. Enrolled participants were randomly allocated to three parallel groups: the bojungikgitang, banhabaekchulchonmatang, and placebo arms. Each participant was examined for signs and symptoms of tinnitus before and after taking medication. A follow-up to evaluate the maintenance of efficacy was performed (Figure 1).

### Recruitment

We recruited participants by advertisements in local newspapers and on the websites of local medical centres in Gyeonggi-province, Korea. Respondents were contacted by clinical trial coordinators (CRC) to determine eligibility via a telephone pre-screening. If an applicant met the study criteria, he or she was invited to the clinical research centre of the Wonkwang Univ. Sanbon Oriental Medical Centre and was examined for eligibility. Oriental medicine doctors, ophthalmologists, and gastroenterologists conducted the screening together to improve the quality of eligibility assessment. Oriental medicine doctors managed the whole process of screening. Ophthalmologists conducted the otologic examination and assessed the brain computed tomography (CT) to identify and exclude conditions including otitis media, acoustic tumours, head trauma, etc. Gastroenterologists assessed the complete blood cell count (CBC), erythrocyte sedimentation rate (ESR), blood chemistry, PA chest film, and urine analysis to exclude patients with serious liver and kidney disease.

### Inclusion criteria

Eligible participants of both sexes who were 19 years and older were enrolled according to the inclusion and exclusion criteria. We defined the term tinnitus as a subjective form of the disorder. Subjective tinnitus is the involuntary perception of the concept of a sound in the absence of an external source [1]. We included participants undergoing typical conditions of intermittent or continuous tinnitus for three or more months. Participants were instructed to discontinue other treatments for tinnitus during the run-in, treatment, and follow-up periods. Written informed consent was obtained from each participant.

### Exclusion criteria

Participants were excluded if they suffered from serious medical conditions, such as uncontrolled hypertension, diabetes mellitus requiring insulin injection, past or current malignancy, liver or kidney dysfunction, anaemia, active pulmonary tuberculosis, or other infectious or systemic diseases incompatible with treatment with herbal medicines.

The exclusion criteria were as follows: administration of other forms of tinnitus treatment; underlying disease or history of otitis media, acoustic tumour, intracranial lesion, inner ear malformation, head trauma, or the use of ototoxic medications; pregnancy, lactation, or the lack of a form of contraception; participation in another clinical trial within one month of enrolment; auditory surgery, a major surgery, or a blood transfusion within one month of enrolment; hypersensitivity or allergic reactions to herbal medicines that are related to this trial; diseases that can affect the absorption of drugs or disordered digestion after surgery related to such a disease; history of a neuropsychiatric abnormality, such as manic-depression, schizophrenia, alcoholism, drug addiction, etc.; inability to understand written consent or to engage in this study due to mental retardation or other emotional or mental problems; or judgment by an expert that the potential subject's participation is inappropriate.

Exclusion was primarily based on the information provided by the patients. Also, additional examinations, such as CBC, ESR, blood chemistry, PA chest film, urine analysis, Brain CT, otologic examination, and vital signs, were performed before the trial to ensure that the patients did not suffer from these diseases.

Over-the-counter (OTC) drugs were allowed for the management of episodic colds, headaches, dyspepsia, etc.; however, participants were gently advised to reduce the doses, stop taking the supplements, or refrain from adding other supplements to their regimen. Some OTC drugs, such as those containing ginkgo or zinc, were not allowed, as there are concerns that these drugs might have direct impacts on tinnitus. These restrictions were based on the information provided by patients, and then the researchers evaluated whether or not some of these components would impact tinnitus.

We recorded the drugs taken by each participant at every visit, and participants were asked to notify us of any changes in their medication/supplement regimen. Additional herbal prescriptions, acupuncture treatments, or therapeutic interventions by other clinicians were not allowed during the trial period. The complete eligibility criteria are in Appendix 1.

### Sample size and randomisation

We wished to estimate the sample size that would be sufficient to detect significant differences in the Tinnitus Handicap Inventory (THI) between the experimental and control groups. Sample size was determined prospectively, as described in a previous study [12]. The mean and standard deviation of the expected difference scores in THI were 6.550 and 6.778 between the experimental and control group. The following formula was used to estimate the sample size for a two-group trial:

Calculations were performed using 80% power, a 5% significance level, and a 25% dropout rate. The required sample size was approximately 22 participants for each group. It was necessary to increase the sample size by 30 per group for parametric estimation. We planned to enrol 120 participants in each of the three groups, allowing for a 25% withdrawal rate.

An expert on statistics who had direct contact with the study participants conducted the randomisation of participants. Participants were assigned random numbers via block randomisation using the SPSS statistical package (Version 12.0; SPSS Inc., Chicago, IL). The block size was concealed from other researchers, and the randomisation table was not available for assessment by anyone else involved in the study.

Opaque sealed envelopes containing serial numbers were delivered to the clinical trial centre. Before the random assignment, all participants were informed that they would be allocated to one of three groups. Random allocation was conducted at the second visit. Random numbers with their corresponding participants were determined in the order of the time of second visit. The allocation results were not announced to the participants until the last visit of the last randomised participant.

The success of blinding was assessed at each participant's last visit. Researchers who were blinded to the allocation results performed the outcome assessment.

### Treatment protocol

Participants received bojungikgitang, banhabaekchulchonmatang, or a placebo-drug for eight weeks. Oral administration occurred according to the following statements:

Patients in group 1 received bojungikgitang (see Additional file 1) and instructions on how to make the tea; they consumed a packet of the medicine (12.52 g) with tepid water three times a day at 30 minutes after meals.

Patients in group 2 received banhabaekchulchonmatang (see Additional file 2) and instructions on how to make the tea; they consumed a packet of the medicine (12.52 g) with tepid water three times a day at 30 minutes after meals.

Patients in group 3 received the placebo medicine (powdered extract), used in the same way as in groups 1 and 2.

The defined daily doses (DDDs) of these herbal medicines should be determined based on the specifications and analytical procedures of drug products in KFDA guidelines. However, no pilot study has been reported so far.

#### Placebo medicine

Hanpoong Pharm and Foods Co., Ltd., produced the placebo medicine according to Korea Good Manufacturing Practice (KGMP) standards. They developed a homogeneous powdered extract of yellow colour, which was made by mixing 1.118 g of cornstarch, 1.118 g of lactose, 6.80 g of Allura red, 1.05 g of Brilliant Blue, and 5.65 g of tartrazine. The colour, form, weight, odour, and taste of the placebo were very similar to the treatment medicines.

### Permitted and prohibited concomitant treatments

Medical supplies for internal use, ointments, and other goods not approved for medical treatment, but approved by the Food and Drug Administration, could be used in based diseases that have nothing to do with tinnitus according to the customs. However, nutraceutical or oriental medicines that have herbal ingredients and are used in healthy people were prohibited.

### Primary outcome measurement

THI was used as the primary outcome variable. The purpose of this questionnaire was to identify difficulties that may be experienced because of tinnitus. It measures tinnitus-related cognitive, emotional, and social problems [13]. The THI consists of three scales: a Functional subscale (eleven factors), an Emotional subscale (nine factors), and a Catastrophic subscale (five factors). It was measured at baseline (one week before treatment initiation), at four weeks after the first medication administration, at eight weeks after the first medication administration, and at follow-up (two weeks after treatment termination).

### Secondary outcome measurement

Secondary outcome measures included the Acoustic Examination (AE) and the Visual Analogue Scale (VAS). Two VASs measuring the perceived loudness of tinnitus and its impact on life were given. The VAS consists of 100-mm lines with endpoints denoted by the words 'total absence' and 'maximum'. It was measured at baseline (one week before treatment initiation), at four weeks after the first medication administration, at eight weeks after the first medication administration, and at follow-up (two weeks after treatment termination). The EuroQoL 5-Dimension (EQ-5D) and the Health Utilities Index Mark 3 (HUI3) were measured at baseline, at eight weeks after the first medication administration, and at follow-up, as a measure of health outcomes. The data collection schedule is detailed in Table 1.

**Table 1:** Data Collection Schedule

Period	Screening	Run-in phase	Treatment period			Follow- up
**Visit**	**1st (D -7)**		**2nd (D 1)**	**3rd (D 29)**	**4th (D 57)**	**5th (D 71)**

Informed Consent	○					

Demographic Characteristics	○					

Medical History	○					

Safety Assessment	○		○	○	○	

Otologic Examination	○					

Acoustic Examination	○			○	○	○

Tinnitus Handicap Inventory	○			○	○	○

Visual Analogue Scale	○			○	○	○

EQ-5D	○				○	○

Health Utilities Index Mark 3	○				○	○

Adverse Effects				○	○	○

Blinding Assessment					○	

### Patient safety

All patients underwent routine testing that included the following: complete blood cell count (CBC), erythrocyte sedimentation rate (ESR), and blood chemistry, as well as PA chest film and urine analysis before randomisation and immediately after completing the treatment. Brain computed tomography (CT) and otologic examination were measured at baseline. Vital signs were measured at every visit, except for the last visit. These tests helped to identify and exclude patients with serious liver and kidney disease as well as those with other severe illnesses. Furthermore, the results of these tests allowed for herbal medication-associated risks.

### Statistical analysis

Analyses were performed for two populations: 1) an intention-to-treat population consisting of all randomised participants who have at least one measurable outcome report following treatment (missing data were replaced with the last observation values) and 2) a per-protocol population including only participants without major protocol deviations. All data were descriptively analysed. All main analyses were based on the intention-to-treat population. For primary and secondary outcome measures, the mean differences from baseline values to the end of treatment were compared using paired T-test, ANOVA (analysis of variance), or MANOVA (Multivariate Analysis of Variance). A trend test was also performed using repeated measures analysis of variance. We adjusted for possible confounders using the criteria for statistical significance.

Statistical analyses were performed using the SPSS statistical package program (ver. 18.0), and the level of significance was established at α = 0.05.

### Data and safety monitoring

Monitoring was conducted for quality control. Investigators could also be convened to discuss practical issues that might be encountered, such as dealing with serious adverse events, revising the protocol, and addressing certain important issues that might be raised by investigators and participants.

We defined adverse events as unintended signs, symptoms, or disease occurring after treatment that were not necessarily related to the intervention. The safety assessment was based primarily on the frequency of adverse events, which included all serious adverse events. Information regarding adverse events was summarised by presenting the number and percentage of participants experiencing any adverse events.

### Ethics

Written consent was obtained from each participant. This study protocol was approved by the institutional review board (IRB) of the Wonkwang University Oriental Medical Centre.

## Abbreviations

AE: Acoustic Examination; ANOVA: analysis of variance; CAM: complementary and alternative medicine; CBC: complete blood cell count; CRC: clinical trial coordinator; CT: computed tomography; DDD: defined daily dose; ESR: erythrocyte sedimentation rate; EQ-5D: EuroQoL 5-Dimension; FDA: Food and Drug Administration; HUI3: Health Utilities Index Mark 3; IRB: institutional review board; KGMP: Korea Good Manufacturing Practice; KHIDI: Korea Health Industry Development Institute; KNHI: Korean National Health Insurance; KNHID: Korean National Health Insurance Data; MANOVA: multivariate analysis of variance; OTC: Over-the-counter; THI: Tinnitus Handicap Inventory; TKM: Traditional Korean Medicine; VAS: Visual Analogue Scale.

## Competing interests

The authors declare that they have no competing interests.

## Authors' contributions

All authors participated in the conception and design of the trial. NKK is the principal investigator of this study. He drafted the protocol. NKK and DHL wrote the final manuscript. JHL, YLO, IHY, ESS, CHL, and DHL contributed to the research design and made critical revisions. All authors read and approved the final manuscript.

## Appendix 1. Eligibility Criteria

Inclusion criteria

• Age greater than 19 years of either sex

• Typical conditions of intermittent or continuous tinnitus:

Duration of more than three months

Involuntary perception of the concept of a sound without the presence of an external source

• Agree not to receive another treatment during the clinical trial period

• Written and informed consent

Exclusion criteria

• Receiving other forms of tinnitus treatments

• Underlying disease or history of disease:

Otitis media

Acoustic tumour

Intracranial lesion

Inner ear malformation

Head trauma

Ototoxic drug medication, etc.

• Pregnant or lactating women

• Women not using contraception

• Participation in other clinical trials within the last month

• Auditory surgery, a major surgery, or a blood transfusion within the last month

• Drug hypersensitivities or allergies

• Diseases that can affect the absorption of drugs or disordered digestion after surgery related to the disease

• History of neuropsychiatric abnormality:

Manic-depression

Schizophrenia

Alcoholism

Drug addiction, etc.

• Inability to understand written consent or participate this study:

Mental retardation

Mental or emotional problems

• Judgment by an expert that the potential subject is inappropriate for participation in the study

## Supplementary Material

Additional file 1 **Bojungikgitang**. Components of bojungikgitang Click here for file

Additional file 2 **Banhabaekchulchonmatang**. Components of banhabaekchulchonmatang Click here for file
