# Supplementary material for: Bojungikgitang and banhabaekchulchonmatang in adult patients with tinnitus, a randomized, double-blind, three-arm, placebo-controlled trial - study protocol
Source: Trials. 2010 Mar 28;11:34. doi: 10.1186/1745-6215-11-34 (PMC2859364; doi:10.1186/1745-6215-11-34)
Supplement: Additional file 1 — Bojungikgitang. Components of bojungikgitang [file 1745-6215-11-34-S1.DOC]

**Additional files**

Additional file 1.

Title: bojungikgitang

Description: Components of bojungikgitang

Per Serving (12.52 g):

Powdered extract of Astragali Radix (an annexed standard)…………… 1.00 g

Powdered extract of White Ginseng (an annexed standard)…………… 1.33 g

Powdered extract of Atractylodes rhizome white (an annexed standard)…………… 1.33 g

Powdered extract of Glycyrrhizae Resina (an annexed standard)…………… 1.33 g

Powdered extract of Angelicae Gigantis Radix (an annexed standard)…………… 0.67 g

Powdered extract of Fraxini Cortex (an annexed standard)…………… 0.67 g

Powdered extract of Cimicifugae Rhizoma (an annexed standard)…………… 0.25 g

Powdered extract of Bupleuri Radix (an annexed standard)…………… 0.25 g

The remainder was cornstarch.
