# Supplementary material for: Bojungikgitang and banhabaekchulchonmatang in adult patients with tinnitus, a randomized, double-blind, three-arm, placebo-controlled trial - study protocol
Source: Trials. 2010 Mar 28;11:34. doi: 10.1186/1745-6215-11-34 (PMC2859364; doi:10.1186/1745-6215-11-34)
Supplement: Additional file 2 — Banhabaekchulchonmatang. Components of banhabaekchulchonmatang [file 1745-6215-11-34-S2.DOC]

Additional file 2.

Title: banhabaekchulchonmatang

Description: Components of banhabaekchulchonmatang

Per Serving (12.52 g):

Powdered extract of Pinelliae Rhizoma (an annexed standard)…………… 2.00 g

Powdered extract of Hordei Fructus Germinatus (an annexed standard)…………… 2.00 g

Powdered extract of Fraxini Cortex (an annexed standard)…………… 2.00 g

(4.0 g as Fraxini Cortex, 160.0 mg as Hesperidin)

Powdered extract of Atractylodes rhizome white (an annexed standard)…………… 1.33 g

Powdered extract of Massa Medicata Fermentata (an annexed standard)…………… 1.33 g

Powdered extract of Atractylodis Rhizoma (an annexed standard)…………… 0.67 g

Powdered extract of Zingiber officinale (an annexed standard)…………… 0.73 g

Powdered extract of Astragali Radix (an annexed standard)…………… 0.33 g

Powdered extract of Ginseng Radix Alba (an annexed standard)…………… 0.67 g

(1.3 g as Ginseng Radix Alba, 2.6 mg as Ginsenoside Rb1)

Powdered extract of Gastrodiae Rhizoma (an annexed standard)…………… 0.67 g

Powdered extract of Polyporus (an annexed standard)…………… 0.13 g

Powdered extract of Alismatis Rhizoma (an annexed standard)…………… 0.33 g

Powdered extract of Zingiberis Rhizoma Siccus (an annexed standard)…………… 0.20 g

Powdered extract of Phellodendri Cortex (an annexed standard)…………… 0.13 g

(0.5 g as Phellodendri Cortex, 3.0 mg as Berberine (Berberine Chloride))
